# Heterogeneity of material structure determines the stationary surface topography and friction

**DOI:** 10.1038/s41598-018-32545-5

**Published:** 2018-09-21

**Authors:** Qiang Li, Lars Voll, Jasminka Starcevic, Valentin L. Popov

**Affiliations:** 1 Berlin University of Technology, Berlin, 10623 Germany; 2 National Research Tomsk State University, Tomsk, 634050 Russia

## Abstract

The character of surface roughness and the force of friction in the stationary state after a sufficiently long run-in process are of key importance for numerous applications, e.g. for friction between road and tire. In the present paper, we study theoretically and experimentally the asymptotic worn state of a bi-phasic material that is arbitrarily heterogeneous in the contact plane, but homogeneous in the direction of the surface normal. Under the assumption of Archard’s wear law in its local formulation, the asymptotic shape is found in the closed integral form. Given the surface profile, the coefficient of friction can be estimated, since the coefficient of friction is known to be strongly correlated with the mean square root value of the surface slope. The limiting surface profiles and the corresponding coefficient of friction are determined as functions of size, relative concentration and wear ratio of the phases. The results of numerical calculations are compared to and validated by experiments carried out on simplified model systems. The main conclusion is that the rms value of the surface slope is not influenced by the characteristic linear size of inclusions and depends solely on the relative concentration of phases, as well as the ratio of their wear coefficients.

## Introduction

Friction and wear occur everywhere in our daily life. One prominent and economically very important example is the contact of a vehicle’s tire with the road. Many important functional properties are determined by the exact nature of this contact, e.g. skid resistance, which is closely related to the driving safety^1,2^. In the last few decades, a lot of experimental and numerical modeling efforts have been undertaken to study the tire-pavement interaction, which led to the development of new measurement devices^3^, improved characterization of surface texture^4^, development of mixed aggregates and asphalt binder^5^, and assessment of environmental factors^6^. A number of tests have shown that the properties of the aggregate, including hardness and wear resistance, as well as the mixture of coarse and fine aggregates have a strong influence on the frictional properties of the pavement surface^7–9^. The tire-road contact is only one example and a typical representative of a broad class of tribological contacts where a relatively stiff rough surface (which, however, is rarely replaced and able to be worn away over time) is in sliding contact with an elastomer. The viscoelastic contact partner also undergoes wear, but this is of lesser importance, either because it is replaced more often or because its surface profile has lesser influence on the frictional properties of the contact (both of which is the case with tires). Due to wear, the surface topography of the stiff contact partner will evolve over time, which, even more importantly, may lead to changes in the coefficient of friction. The coefficient of friction between a rough rigid substrate and an elastomer is known to depend on a number of system and loading parameters including sliding velocity, temperature, normal load, etc^10^. However, under conditions relevant for most practical applications (corresponding to the region of the long plateau of the coefficient of friction as a function of velocity) it can be loosely estimated as the root mean square (RMS) of the surface gradient^11,12^. In the present paper, we will therefore consider the root mean square value of the surface gradient (surface slope) as a parameter roughly characterizing friction coefficient.

**Figure 1:**
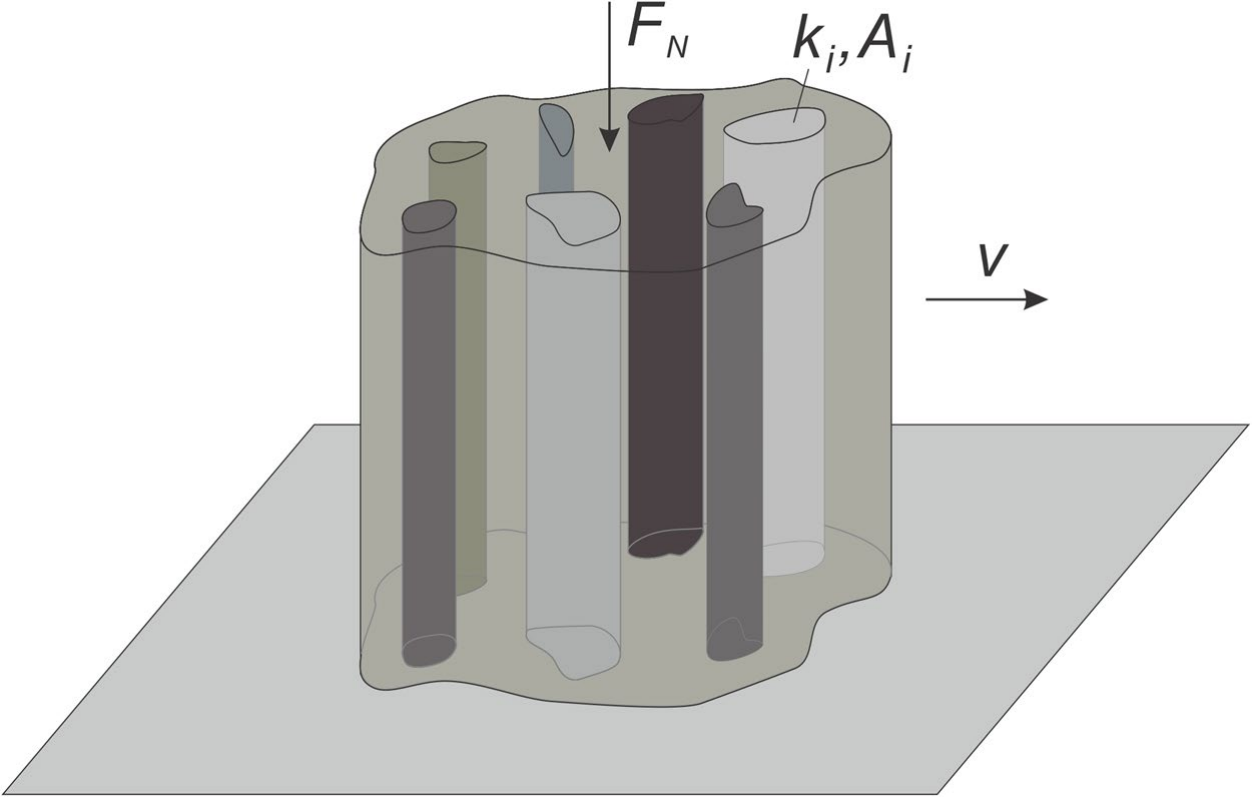
A punch with heterogeneous phases sliding on an elastic half-space.

The system we study in the present paper is schematically shown in Fig. 1. It represents a cylindrical punch with multiphasic composition sliding horizontally with velocity *v* on an elastic half space in the presence of a normal load *F*_*N*_. Similar problems have been studied in the past using the finite element method^13,14^, the discrete element method^15^ and molecular dynamics^16^. However, if we are only interested in the *limiting* shape, solution can be obtained in closed integral form, which enables extensive parameter studies.

## Results

We consider a cylindrical punch composed of a multiphasic material, whose phases *i* have the wear coefficients *k*_*i*_ (defined by eq. (2)) and the cross-sectional areas *A*_*i*_. Let us denote the initial shape of the indenter face as *f*_0_(*x*, *y*). To achieve a qualitative understanding of the wear process, we make the following simplifying assumptions: (a) only the indenter is subject to wear, (b) the elastic properties of all phases are equal, while their wear coefficients may have arbitrary ratios, (c) wear is governed by Archard’s law in its local form, which means that the depth change d*f* at the coordinate (*x*, *y*) due to wear is proportional to the local pressure *p*(*x*, *y*) and the sliding distance d*s*, and is inversely proportional to the material hardness *σ*_0_

1 $${\rm{d}}f(x,y)=\frac{{k}_{wear}}{{\sigma }_{0}}p(x,y)\,{\rm{d}}s.$$

*k*_*wear*_ is a constant that depends on the material couple and is called the wear coefficient. Introducing the notation *k* = *k*_*wear*_/σ_0_, we can rewrite (1) in the form d*f* (*x*, *y*) = *kp* (*x*, *y*) d*s*. For a multiphasic indenter, this equation is valid for each particular phase: d*f*_*i*_ (*x*, *y*) = *k*_*i*_*p*_*i*_ (*x*, *y*) d*s*. Dividing by the time increment d*t*, the linear wear *rate* (wear depth per unit time) is obtained:

2 $$\frac{{\rm{d}}{f}_{i}(x,y)}{{\rm{d}}t}={k}_{i}{p}_{i}(x,y)v.$$

The steady state is achieved when the linear wear rate at all points of the indenter is the same. This means that the pressure inside the area of each particular phase reaches a constant value, which we just continue to denote *p*_*i*_ = *p*_*i*_ (*x*, *y*) (despite the fact that the pressure no longer depends on the coordinates *inside* one phase; it is still a function of coordinates in the sample as a whole), while pressures in different phases fulfil the condition:

3 $${k}_{1}{p}_{1}=\ldots ={k}_{i}{p}_{i}=\ldots ={k}_{n}{p}_{n}=C.$$

Thus,

4 $${p}_{i}=\frac{C}{{k}_{i}}.$$

The constant *C* can be determined from the equation for the total normal force

5 $${F}_{N}=\sum _{j=1}^{n}{p}_{j}{A}_{j}=C\,\sum _{j=1}^{n}\frac{{A}_{j}}{{k}_{j}}$$

leading to

6 $$C=\frac{{F}_{N}}{\sum _{j=1}^{n}\frac{{A}_{j}}{{k}_{j}}}.$$

The pressures in particular phases are now determined with (4) in explicit form

7 $${p}_{i}={F}_{N}\frac{1}{{k}_{i}\sum _{j=1}^{n}\frac{{A}_{j}}{{k}_{j}}}.$$

With this pressure distribution, the normal deformation of the elastic half space can be calculated as^11^

8 $$u(x,y)=\frac{1}{\pi {E}^{\ast }}{\iint }_{A}\frac{p(x^{\prime} ,y^{\prime} )}{r}\,{\rm{d}}x^{\prime} {\rm{d}}y^{\prime} ,$$

where E^*^ = *E*/(1 − *ν*^2^) is the effective modulus of elasticity of the elastic half-space, *E* is its Young’s modulus, *ν* is its Poisson ratio, and $r=\sqrt{{(x-x^{\prime} )}^{2}+{(y-y^{\prime} )}^{2}}$ is the in-plane distance between the points (*x*, *y*) and (*x*′, *y*′). As we consider the indenter to be much stiffer than the counter-body, the deformation of the latter is equal to the worn shape of the indenter. Thus, the limiting worn profile is given by

9 $${f}_{\infty }(x,y)=\sum _{i=1}^{n}(\frac{1}{\pi {E}^{\ast }}{\iint }_{{A}_{i}}\frac{{p}_{i}}{r}\,{\rm{d}}x^{\prime} {\rm{d}}y^{\prime} )=\frac{{F}_{N}}{\pi {E}^{\ast }\sum _{j=1}^{n}\frac{{A}_{j}}{{k}_{j}}}\sum _{i=1}^{n}(\frac{1}{{k}_{i}}{\iint }_{{A}_{i}}\frac{1}{r}\,{\rm{d}}x^{\prime} {\rm{d}}y^{\prime} ).$$

This equation provides the final worn profile in explicit integral form, so that both the profile and all parameters depending on the profile (as e.g. the rms value of the slope) can be calculated very effectively.

### Limiting worn profile of a homogeneous cylinder

Let us start consider some specific examples. We begin with the simplest case of a monophasic cylinder with radius *a* and wear coefficient *k*. In the final state, the pressure over the whole contact will be constant and equal to $\bar{p}={F}_{N}/{(\pi a)}^{2}$. The integral (8) has, in this case, the known analytical solution^11,17^

10 $${f}_{\infty }(r)=\frac{4\bar{p}a}{\pi {E}^{\ast }}\,{\rm{E}}(\frac{r}{a}),\,r\le a.$$

Here *r* is the polar coordinate and E(*k*) denotes the complete elliptical integral of the second kind

11 $${\rm{E}}(k)\,:\,={\int }_{0}^{\pi /2}\sqrt{1-{k}^{2}\,{\sin }^{2}\phi }\,{\rm{d}}\phi .$$

**Figure 2:**
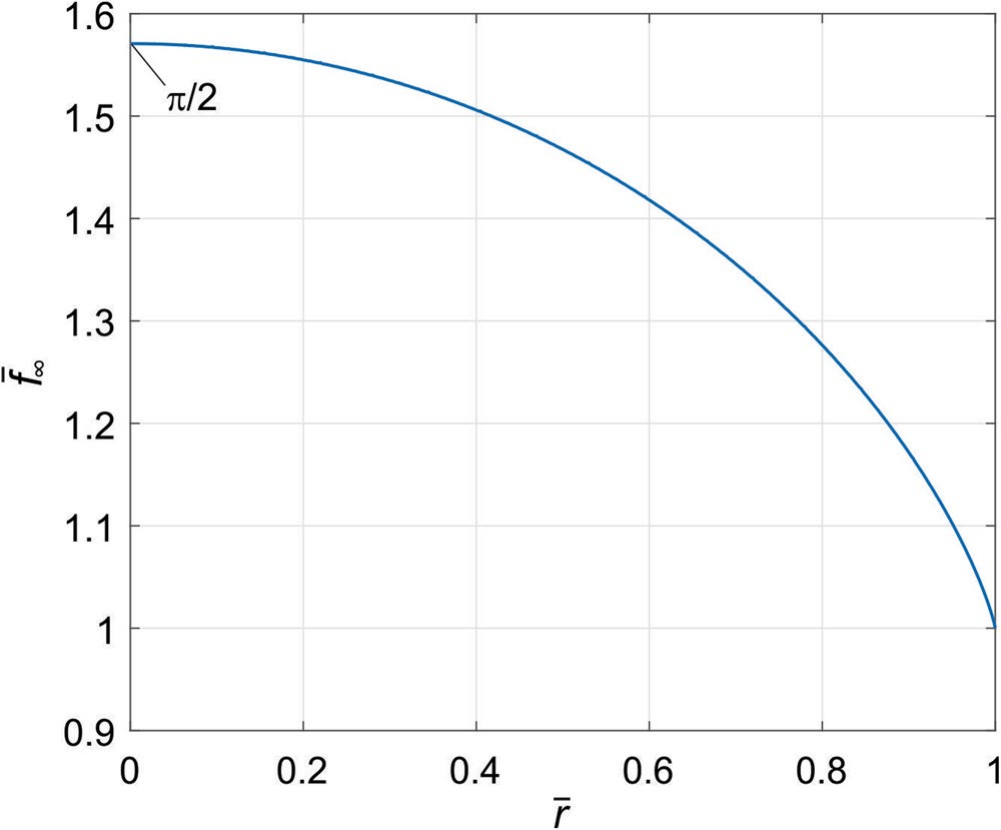
Limiting worn profile of a homogeneous cylinder.

The limiting profile (10) in the dimensionless variables ${\bar{f}}_{\infty }=\frac{{f}_{\infty }}{a}\frac{\pi {E}^{\ast }}{4\bar{p}}$ and $\bar{r}=r/a$ is shown in Fig. 2. The rms value of the surface gradient of this profile is equal to

12 $$\nabla {f}_{\infty }^{0}=\sqrt{\frac{1}{A}{\iint }_{A}{(\frac{{\rm{d}}{f}_{\infty }}{{\rm{d}}r})}^{2}\,{\rm{d}}A}=1.0109\frac{4}{\pi }\frac{\bar{p}}{{E}^{\ast }}\approx \frac{4}{\pi }\frac{\bar{p}}{{E}^{\ast }}$$

Note that neither the limiting shape nor the rms slope depend on the wear coefficient, and are completely determined by the average pressure in the contact and the effective elastic modulus.

### Limiting worn profile of a biphasic cylinder

**Figure 3:**
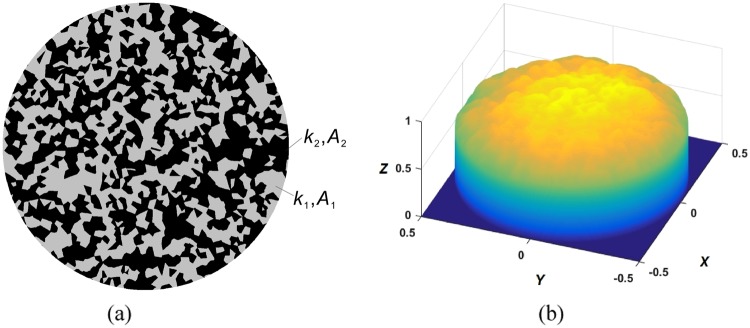
(**a**) An example of a biphasic material with wear coefficients of phases *k*_1_, *k*_2_ and areas *A*_1_ (gray), *A*_2_ (black); (**b**) corresponding surface topography in the stationary state for *k*_2_/*k*_1_ = 10.

In the following, we consider bi-phasic indenters. We continue to consider a cylindrical sample with radius *a*, which, however, is now composed of two different materials: phase 1 having the wear coefficient *k*_1_ and area *A*_1_, and phase 2 with *k*_2_ and *A*_2_ (an example of a typical phase distribution considered in this paper is shown in Fig. 3a; the phases are marked with black and grey colors). Let us introduce the “fill factors” of phases

13 $${\rho }_{1}=\frac{{A}_{1}}{{A}_{1}+{A}_{2}},\,{\rho }_{2}=1-{\rho }_{1}=\frac{{A}_{2}}{{A}_{1}+{A}_{2}}.$$

With this notation, the final surface topography according to (9) is

14 $${f}_{\infty }(x,y)=\frac{1}{{\rho }_{1}{k}_{2}+{\rho }_{2}{k}_{1}}\cdot \frac{\bar{p}}{\pi {E}^{\ast }}({k}_{2}{\iint }_{{A}_{1}}\frac{1}{r}\,{\rm{d}}x^{\prime} {\rm{d}}y^{\prime} +{k}_{1}{\iint }_{{A}_{2}}\frac{1}{r}\,{\rm{d}}x^{\prime} {\rm{d}}y^{\prime} )$$

where $\bar{p}$ is the average pressure in contact. Taking into account (12), one can introduce a normalized shape

15 $$\frac{{f}_{\infty }(x,y)}{\nabla {f}_{\infty }^{0}}=\frac{1}{4}\frac{1}{{\rho }_{1}{k}_{2}+{\rho }_{2}{k}_{1}}\cdot ({k}_{2}{\iint }_{{A}_{1}}\frac{1}{r}\,{\rm{d}}x^{\prime} {\rm{d}}y^{\prime} +{k}_{1}{\iint }_{{A}_{2}}\frac{1}{r}\,{\rm{d}}x^{\prime} {\rm{d}}y^{\prime} )$$

which does not depend on the normal force and elastic properties of the contacting bodies and is completely determined by the geometry of the phase distribution and the ratio of wear coefficients.

**Figure 4:**
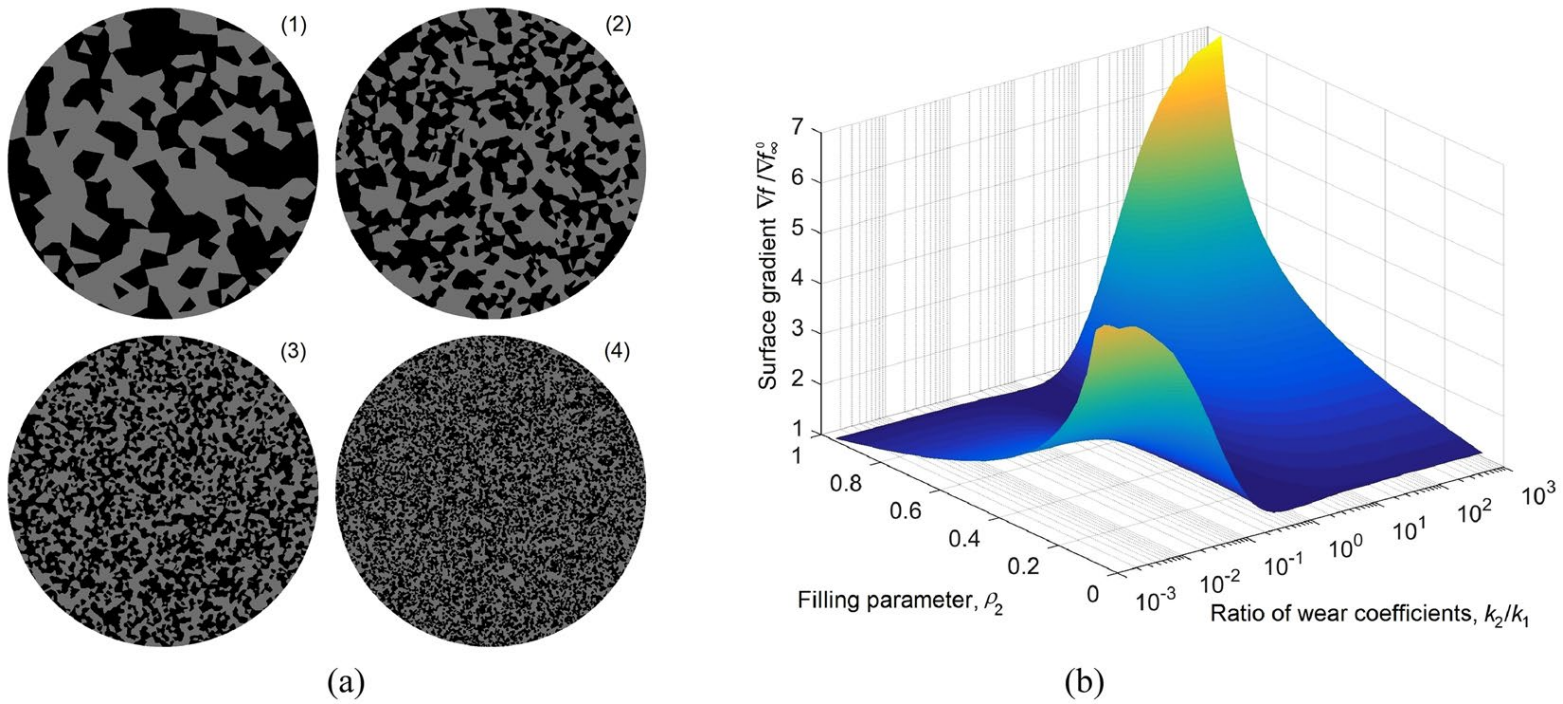
(**a**) Four samples of biphasic materials with the same *ρ*_2_ = 0.5 but different size of ‘cells’; (**b**) dependence of the surface gradient on the density *ρ*_2_ and the ratio of wear coefficients *k*_2_/*k*_1_.

**Figure 5:**
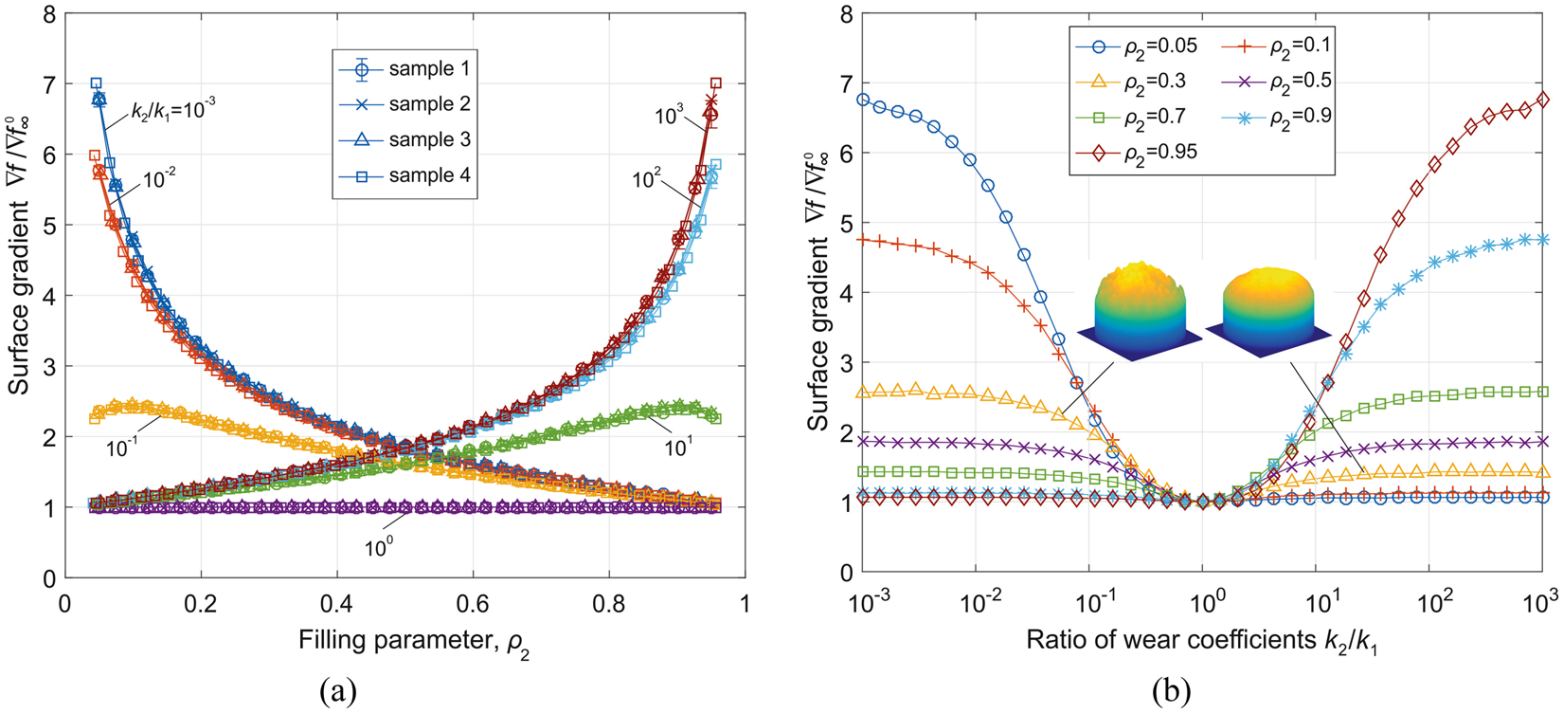
Detailed dependence of the RMS of surface gradient (**a**) on the concentration of phase 2 for varying ratios of the wear coefficient; (**b**) on the ratio of wear coefficients for varying phase concentration.

The generation of phase distributions and numerical evaluation of the integrals (15) is described in the “Methods” section. An example phase distribution and the resulting limiting worn profile are shown in Fig. 3a,b correspondingly. The main target parameter of our study is the rms value of the surface slope, ∇*f*. Let us start by elucidating the role of scaling in the formation of the limiting value of ∇*f*. For this sake, we generated four samples with the same statistical properties of phase distribution but different characteristic scale, as shown in Fig. 4(a) for the case of *ρ*_1_ = *ρ*_2_ = 0.5. Similar structures have been produced for fill factors *ρ*_2_ varying linearly from 0.05 to 0.95 and for a number of ratios of wear coefficients, *k*_2_/*k*_1_, varying logarithmically from 10^−3^ to 10^3^. The value of the rms slope for each set of parameters was averaged over 10 random realizations of the phase distribution. The results of these simulations are shown in Fig. 4b, where the normalized rms slope $\nabla f\,/\,\nabla {f}_{\infty }^{0}$ is plotted. Figure 5a, shows detailed dependencies of the rms slope on the fill factor *ρ*_2_ for five different values of the ratio of wear coefficients and for all four samples with different coarseness of phase distribution (which are marked with different symbols). One can see that the points corresponding to the same values of wear ratio but different coarseness collapse onto a common curve, thus clearly showing that the scaling has no influence on the rms slope. Therefore, in Fig. 5b we only show the results for the sample 2 as a representative of structures with arbitrarily coarse grain.

Thus, the asymptotic rms slope is a function of only two parameters: the fill factor (e.g. *ρ*_2_) and the ratio of wear coefficients *k*_2_/*k*_1_. It is clear that this dependency remains invariant by transformation *ρ*_1_ →*ρ*_2_, *k*_1_/*k*_2_ → *k*_2_/*k*_1_, which just changes notation without changing the configuration of the contact: $\nabla f({\rho }_{2},{k}_{2}/{k}_{1})=\nabla f(1-{\rho }_{2},{k}_{1}/{k}_{2})$. From the overview in Fig. 4(b), one can see that the largest values of the rms surface gradient (and thus coefficient of friction) correspond to the case when both *ρ*_2_ and *k*_2_/*k*_1_ are large or when both are small. In other words, large final rms slopes correspond to structures with a small fill factor of the phase with the smaller wear coefficient. In this case, the low-wear phase represents a dilute solution of particles in an easily worn matrix. In this extreme case the resulting surface topography resembles a set of “pillars”. It is intuitively clear that such a brush-like structure will have a large coefficient of friction. In the other extreme case of large concentration of the low-wear phase, the worn surface will consist almost entirely of this phase with only small number of shallow “dimples”, where the softer inclusions have been worn away. Two surface topographies with the same fill factor *ρ*_3_ = 0.3 but different ratios of wear coefficients (*k*_2_/*k*_1_ < 1 and *k*_2_/*k*_1_ > 1 correspondingly) are shown in the insert of Fig. 5b.

Let us take a closer look at the extreme “pillars” case, assuming that

16 $${\rho }_{1}{k}_{2}\ll {\rho }_{2}{k}_{1}\,{\rm{and}}\,{k}_{2}{\iint }_{{A}_{1}}\frac{1}{r}\,{\rm{d}}x^{\prime} {\rm{d}}y^{\prime} \ll {k}_{1}{\iint }_{{A}_{2}}\frac{1}{r}\,{\rm{d}}x^{\prime} {\rm{d}}y^{\prime} .$$

In this case, the profile (14) takes the form

17 $${f}_{\infty }(x,y)=\frac{\bar{p}}{\pi {E}^{\ast }{\rho }_{2}}{\iint }_{{A}_{2}}\frac{1}{r}\,{\rm{d}}x^{\prime} {\rm{d}}y^{\prime} ={f}_{\infty }(x,y)=\frac{{p}_{2}}{\pi {E}^{\ast }}{\iint }_{{A}_{1}}\frac{1}{r}\,{\rm{d}}x^{\prime} {\rm{d}}y^{\prime} ,$$

where we used relation $\bar{p}\approx {p}_{2}{\rho }_{2}$ which, under the above assumptions, follows from (7). Assuming for simplicity that the hard inclusions have a circular cross-section, we can find the rms slope by immediate use of eq. (12)

18 $$\nabla {f}_{\infty }\approx \frac{4}{\pi }\frac{{p}_{2}}{{E}^{\ast }}=\frac{4}{\pi }\frac{\bar{p}}{{E}^{\ast }{\rho }_{2}}=\frac{\nabla {f}_{\infty }^{0}}{{\rho }_{2}}.$$

In this limiting case, the rms slope does not depend any more on the ratio of wear coefficients and is inversely proportional to the fill factor of the hard phase. This asymptote can be easily seen in Fig. 5(a) for *k*_2_/*k*_1_ = 10^−3^. Note that the rms gradient depends only on the overall fill factor, but not on the radius of the inclusions, which is consistent with the lack of scale-dependence found above in the study of random configurations. We thus come to the conclusion that the best way to maintain a high coefficient of friction in the stationary worn state is to use a matrix with a small fraction of hard (low-wear) inclusions. As an example, consider the problem mentioned in the introduction – wear and friction in the tire-road contact. Assuming that the deformation of tire in the contact area is of the order of magnitude of 8%, and thus $\bar{p}\,/\,{E}^{\ast }\approx 0.08$, a stationary rms slope of the order of unity will be achieved with *ρ*_2_ ≈ 0.1. Thus, for producing a stationary coefficient of friction on the order of unity, the area fill factor of hard inclusions should be of the order of 10%.

### Experimental results

To experimentally validate the results of our numerical simulations, we investigated the worn shape of homogeneous and heterogeneous cylinders. We did not aim to carry out comprehensive parameter studies as in the foregoing theoretical analysis, and confined ourselves to three model systems, which we consider to be representative generic cases: (i) a homogeneous sample, (ii) high-wear matrix with low-wear inclusions, and (iii) low-wear matrix with high-wear inclusions.

**Figure 6:**
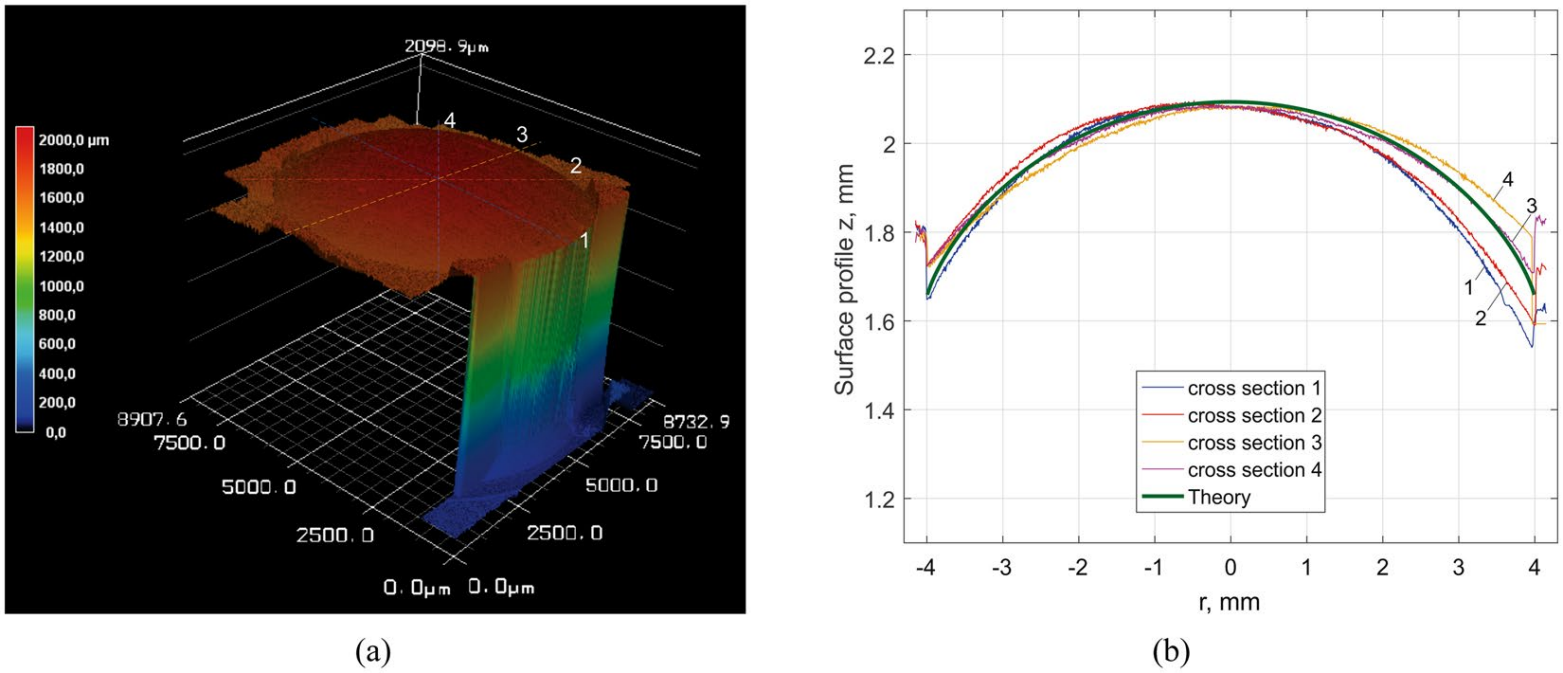
(**a**) Worn surface of an epoxy cylinder after sliding on an elastomer. (**b**) Profile of cross sections in comparison with theoretical solution (calculated with E^*^ = 1.3 MPa).

We first studied wear of a homogeneous cylinder made of epoxy with a radius *r* = 4 mm. The cylinder was pressed against a rubber band with the normal load *F*_*N*_ = 10 N and pulled back and forth with a constant velocity *v* = 0.01 m/s. The worn surface, measured with a scanning laser interference microscope (see Section “Methods”), is shown in Fig. 6a, and the profiles of four cross sections, marked in Fig. 6(a) with numbers 1 to 4, are shown in Fig. 6b. For comparison, the theoretical solution given by equation (10) is shown in the same plot (green line). It is seen that the theoretical curve is quite close to the experimentally obtained profiles. The small asymmetry is likely the result of a slightly inclined sample.

**Figure 7:**
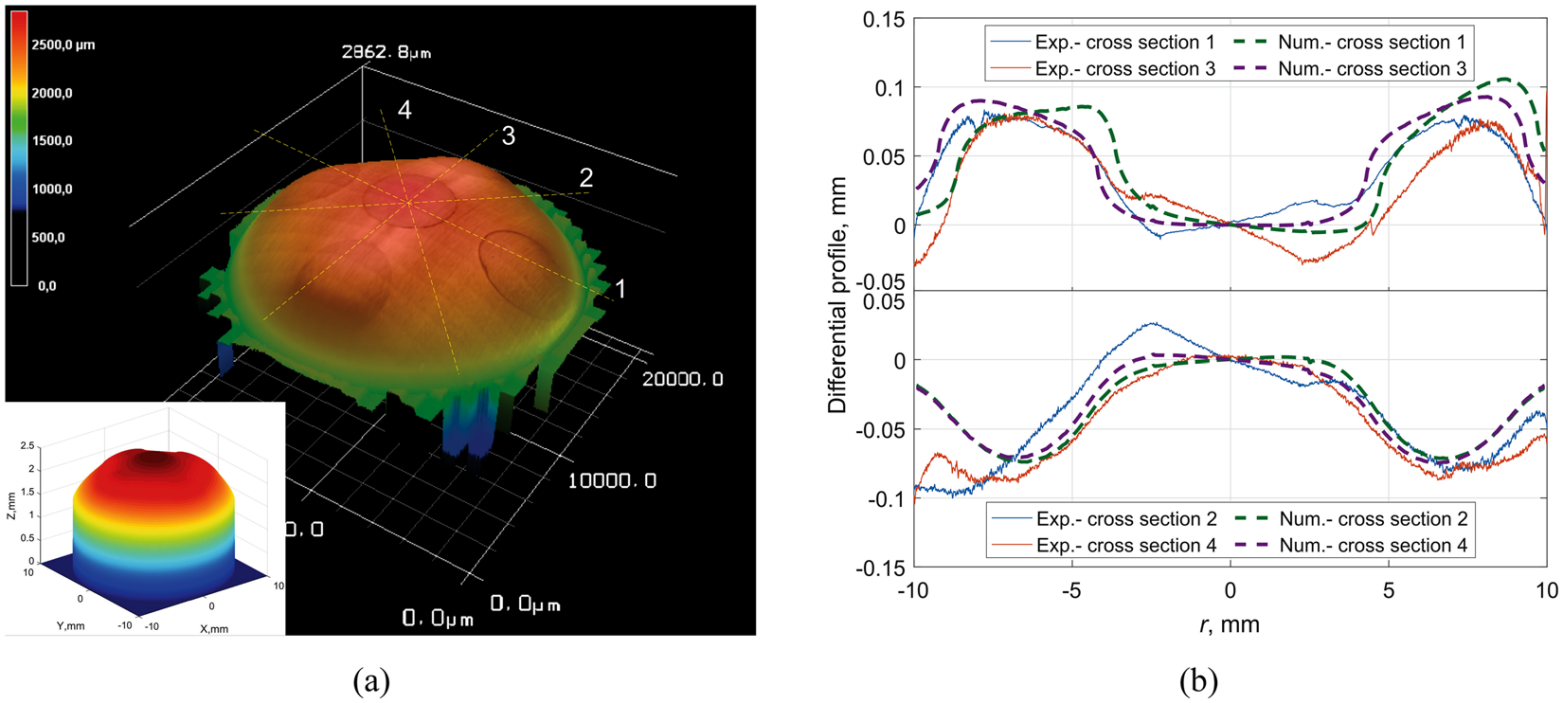
(**a**) Worn surface of biphasic cylinder (steel rods in brass matrix) due to wear in contact with an elastomer. (**b**) Differential profile of cross sections in comparison with numerical results.

**Figure 8:**
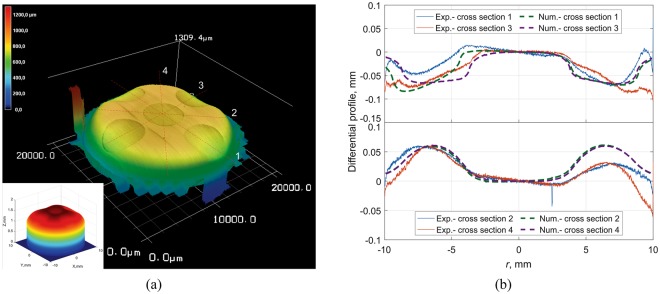
(**a**) Worn surface of biphasic cylinder (brass rods in steel matrix) due to wear in contact with an elastomer. (**b**) Differential profile of cross sections in comparison with numerical results.

In the next step we prepared bi-phasic brass-steel cylinders. In Figs 7 and 8, one can see the final worn profiles of bi-phasic cylinders consisting of five rods with radius *r* = 2.5 mm embedded in a cylinder with radius *r* = 10 mm: Fig. 7 shows the results for brass rods in a steel matrix and Fig. 8, correspondingly, steel rods in a brass matrix. In Fig. 7a, the steel rods are less worn and produce visible protrusions. In the case of brass in steel, the rods are worn more intensively and clearly result in the formation of dimples (Fig. 8a). The numerical results with parameters *F*_*N*_ = 35 N and *k*_steel_/*k*_brass_ = 1.7 for both cases are shown in the corresponding subplots. The elastic modulus of the elastic counterpart was assumed to be E^*^ = 1.3 MPa in all cases, as determined from experiments with homogeneous cylinders. To allow for a more detailed comparison of the effects of heterogeneity on the worn profile, in Figs 7b and 8b *differential profiles* of four cross sections are shown. Differential profiles were determined as the difference between the profile of some cross section and the “averaged profile” obtained by averaging the surface height over ring segments at any given radius *r*. In this way, the macroscopic curvature of the sample is subtracted and the resulting profile shows more clearly the effect of the phase heterogeneity (for a homogeneous material the differential profile would be approximately constant.) It can be observed that the experimental findings match theoretical predictions reasonably well in both cases of steel rods in brass and brass rods in steel. A possible cause for the observed small discrepancies between experimental findings and theoretical results could be the use of the glued abrasive paper, while in simulation we assumed the counter body to be a homogeneous elastomer.

## Discussion

Under the assumption of Archard’s wear law, we proposed a method for calculating the surface topography of a multiphasic punch in the stationary worn state. The limiting shape of the body after a long run-in process has been obtained in explicit integral form. The evaluation of the integrals was carried out using an in-house BEM code described in^18^. The obtained solution is applicable to systems with an arbitrary number of different phases and arbitrary geometrical in-plane configuration of the phases. A detailed numerical study was carried out only for the case of bi-phasic indenters.

Using the obtained limiting profiles, the rms slope has been determined as a topographic property most directly related to the coefficient of friction. Both simulations and analytical estimations show that the limiting shape does not depend on the coarseness of the phase structure. Thus, compositions that only differ in coarseness of the grain will have the same limiting rms slope. The complete set of parameters determining the final state and the limiting rms slope was determined to be: average (apparent) pressure in the contact area, fill factors (area fractions) of the phases, and the ratio of the wear coefficients. The largest limiting rms slope is obtained in the case of a small fraction of hard (low-wear) fibers, which leads in the final state to a “pillar-like” structure. In the case of a composite with a small concentration of very hard inclusions, the final rms slope is roughly inversely proportional to the area fill factor of the hard phase. The obtained result provides a rule for the design of composite structures with specified frictional properties after run-in.

For validation of the underlying contact mechanics, we carried out experiments on homogeneous and heterogeneous samples and compared the results with theoretical predictions. The experimental results show very good qualitative and acceptable quantitative agreement with theoretical predictions. They confirm that the use of Archard’s law of wear in its local formulation is a sensible assumption, allowing correct understanding of wear of composite structures.

It should be stressed that the above results are not universal, and rely on a number of assumptions: (1) The wearable composite contact partner (cylindrical punch in Fig. 1) was assumed to be heterogeneous in the contact plane but homogeneous in the z-direction (depth), effectively consisting of a bundle of fibers or rods oriented normal to the surface; (2) the normal load is kept constant during the wear process; (3) the sliding velocity is assumed to be constant over the entire contact area. This means that that gross sliding is assumed. However, the actual velocity is not relevant and may even vary in time; (4) the counter-body is considered to be elastic and either non-wearable or regularly replaced; (5) finally we assume the validity of Archard’s wear law in a local form. However, the results will be similar for any *local* wear law that is not necessarily linear in normal load.

The assumption (4) may look strange at first glance, but it was chosen with the motivation of pavement wear due to contact with tires described in the introduction. With regard to elasticity, the road can be considered rigid, while tires have significant elasticity. With regard to wear, the situation is the opposite: We assume that only the road is worn, and tires not. With the described conceptual background, it is not important, whether the tires wear or not, as they are changed out regularly, while the road remains the same over long intervals of time. In the experiments, we sought to give our model system the same conceptual properties: a rigid and wearable sample and soft and replaceable counter body.

In real systems one or many of these assumptions may be violated. Thus, the assumption (1) (cylindrical geometry) is of course very artificial. In real composites such as asphalt pavement the composite components have a general three-dimensional geometry. For such geometry, a stationary state will never be reached, since some inclusions are worn down completely, while others become exposed. However, if the ratio of the wear coefficients is not too large, we expect that the worn state will be very similar to that described in the present paper at any given time, although the exact statistical realization of the topography will change slowly with progressive wear. However, if the ratio of wear coefficients is very large, then the system with three dimensional inclusions will be far from the stationary state described here. For such systems, additional analysis, probably using different numerical approaches, would be needed. With respect to the wear law, the results are more robust. As a matter of fact, the only required assumption used in the paper is that the wear rate is local and depends only on pressure. The pressure-dependence can be nonlinear, but if the wear law is non-local (e.g. we consider the finite size of wear particles), then the results will be violated and additional analysis will again be needed. Since the wear law tends to be strongly system-dependent, one cannot make a more general statement of the range of validity of the presented results. The present study has thus to be considered as a mathematical model under some strict conditions, which have to be checked for particular applications.

## Methods

### Boundary Element Method

The final surface topography (9) was calculated using the Boundary Element Method (BEM), with the given pressure distribution (7). This Method is based on the evaluation of integrals of the type of (8) using a fast convolution based on the Fast Fourier Transform^18–20^. In the present contact problem, the evaluation of integrals (14) was implemented as a module for our in-house BEM code.

### Generation of surface patterns

The biphasic surface patterns have been generated using the method of Voronoi diagrams, which has many practical applications in various scientific fields, such as geometry, biology, meteorology, architecture, etc.^21^. In this algorithm, seed points are distributed randomly in a plane, after which cells are formed around each seed by assigning every point in the plane to its closest seed point. In the four samples of Fig. 4a, cells with a total number of 20^2^, 40^2^, 80^2^ and 160^2^ points have been generated. Afterwards, seeds were randomly labelled as phase 1 or phase according to the defined fill factor. The “average diameter” of the cells in the four samples is approximately 1, 1/2, 1/4 and 1/8 relative to each other. The produced structures can be considered self-similar under magnification.

### Experimental investigation

**Figure 9:**
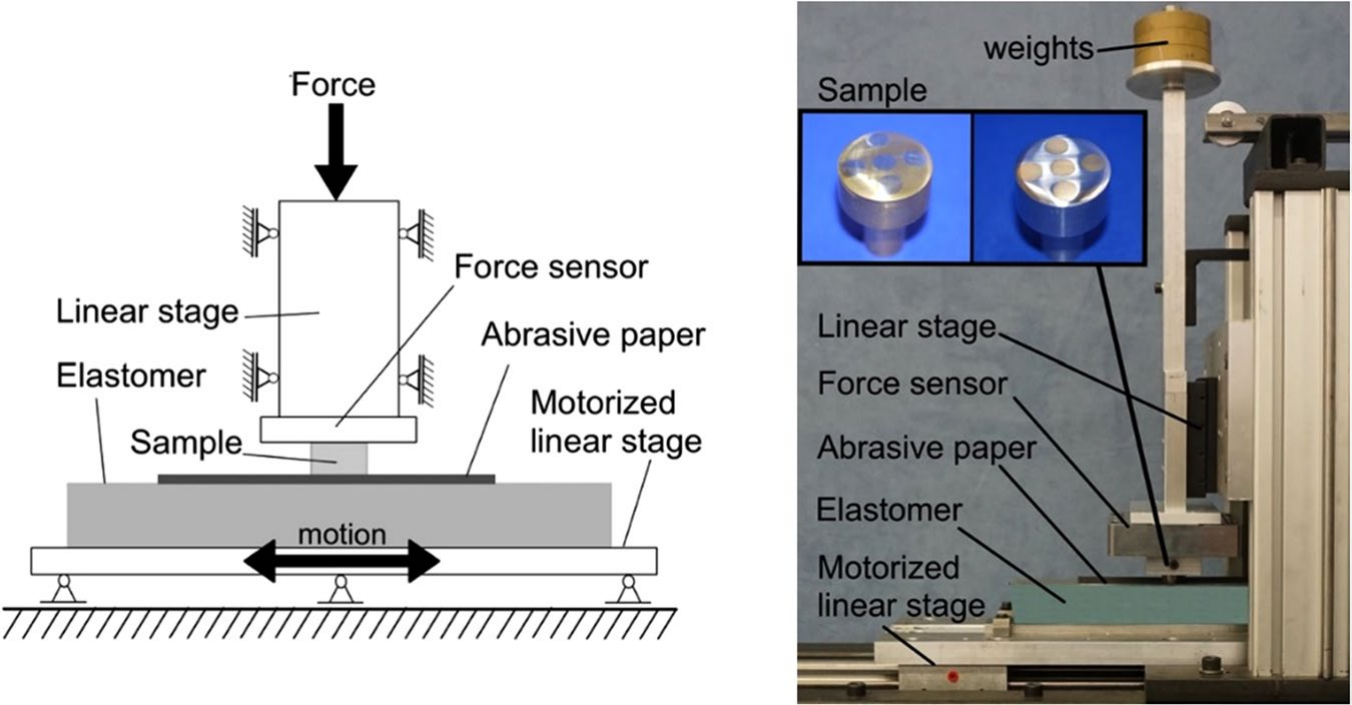
Experimental set-up for wear contact between a cylinder and a rubber block covered by a sandpaper.

The experiment was carried out with the experimental set-up shown in Fig. 9. The rubber block with a size of 380 × 40 × 40 mm was glued to a linear stage that moved horizontally with controlled velocity. The material of the band is polyurethane with elastic modulus of about 1.3 MPa. The indenter was fixed on another linear stage that could move vertically. The normal load was measured by a force sensor placed between the actuator and the sample. To increase and precisely control the wear rate, abrasive fine sandpaper was glued to the rubber band.

The indenter was pressed into the rubber band with a fixed load; subsequently, the linear stage moved horizontally with a constant velocity 0.01 m/s. After 50 times of back and forth linear movement, the sandpaper was replaced to provide constant wear conditions. The orientation of the indenter remained unchanged during the whole experiment.

### Measurement of surface topography

The topography of the worn samples was measured with the confocal 3D laser scanning microscope VKX150/160 of Keyence. This microscope uses a laser light receiving element with 16 bit PMT, a color CCD image sensor with a recording resolution of 3072 × 2304 pixels and confocal optics with pinhole. A single recorded image has a size of 168 × 126 μm with a height resolution of 5 nm. To measure the entire sample 340 images were taken and merged them to a single image 20 × 20 mm in size with the help of the “Auto Stage for intelligent xy control-program”. Photos of the two worn samples that were measured in Figs 7 and 8 are shown in Fig. 9.

## References

[1] Hall, J.W. *et al*. *Guide for Pavement Friction Contractor’s Final Report for National Cooperative Highway Research Program Project* 01–43, *Transportation Research Board of the National Academies*, Washington, DC (2009).

[2] Wallman, C.-G., Åström H. *Friction Measurement Methods and the Correlation Between Road Friction and Traffic Safety – A Literature Review Report of the Swedish National Road and Traffic Institute*, VTI meddelande 911A, Linköping, Sweden (2001).

[3] Andriejauskas, T., Vorobjovas, V. & Mielonas, V. Evaluation of skid resistance characteristics and measurement methods. In *The 9th International Conference ‘Environmental Engineering’* (2014).

[4] Dunford, A. Friction and the texture of aggregate particles used in the road surface course. (University of Nottingham, 2013).

[5] Sarang G, Lekha BM, Geethu JS, Shankar AUR (2015). Laboratory performance of stone matrix asphalt mixtures with two aggregate gradations. J. Mod. Transp..

[6] Do M, Cerezo V, Beautru Y, Kane M (2014). Influence of Thin Water Film on Skid Resistance. J. Traffic Transp. Eng..

[7] Kogbara RB, Masad EA, Kassem E, Scarpas A, Anupam K (2016). A state-of-the-art review of parameters influencing measurement and modeling of skid resistance of asphalt pavements. Constr. Build. Mater..

[8] Zhang X, Liu T, Liu C, Chen Z (2014). Research on skid resistance of asphalt pavement based on three-dimensional laser-scanning technology and pressure-sensitive film. Constr. Build. Mater..

[9] Asi IM (2007). Evaluating skid resistance of different asphalt concrete mixes. Build. Environ.

[10] Popov VL, Voll L, Kusche S, Li Q, Rozhkova SV (2016). Generalized master curve procedure for elastomer friction taking into account dependencies on velocity, temperature and normal force. Tribol. Int..

[11] Popov, V. L. *Contact Mechanics and Friction. Physical Principles and Applications*, 2^nd^ Edition (Springer, 2017).

[12] Grosch KA (1963). The Relation between the Friction and Visco-Elastic Properties of Rubber. Proc. R. Soc. London A.

[13] AbuBakar AR, Ouyang H (2008). Wear prediction of friction material and brake squeal using the finite element method. Wear.

[14] Gallego L, Nélias D, Deyber S (2010). A fast and efficient contact algorithm for fretting problems applied to fretting modes I, II and III. Wear.

[15] Champagne M, Renouf M, Berthier Y (2014). Modeling Wear for Heterogeneous Bi-Phasic Materials Using Discrete Elements Approach. J. Tribol..

[16] Aghababaei R, Warner DH, Molinari JF (2016). Critical length scale controls adhesive wear mechanisms. Nat. Commun..

[17] Popov, V. L., Hess, M. & Willert, E. *Handbuch der Kontaktmechanik. Exakte Lösungen axialsymmetrischer Kontaktprobleme*. (Springer, 2018).

[18] Pohrt R, Li Q (2014). Complete Boundary Element Formulation for Normal and Tangential Contact Problems. Phys. Mesomech..

[19] Pohrt R, Popov VL (2015). Adhesive Contact Simulation of Elastic Solids Using Local Mesh-Dependent Detachment Criterion in Boundary Elements Method. Facta Univ. Ser. Mech. Eng..

[20] Popov VL, Pohrt R, Li Q (2017). Strength of adhesive contacts: Influence of contact geometry and material gradients. Friction.

[21] Aurenhammer F (1991). Voronoi Diagrams — A Survey of a Fundamental Data Structure. ACM Comput. Surv..

